# Residential Exposure to Polychlorinated Biphenyls and Organochlorine Pesticides and Risk of Childhood Leukemia

**DOI:** 10.1289/ehp.0900583

**Published:** 2009-01-27

**Authors:** Mary H. Ward, Joanne S. Colt, Catherine Metayer, Robert B. Gunier, Jay Lubin, Vonda Crouse, Marcia G. Nishioka, Peggy Reynolds, Patricia A. Buffler

**Affiliations:** 1 Division of Cancer Epidemiology and Genetics, National Cancer Institute, National Institutes of Health, Department of Health and Human Services, Bethesda, Maryland, USA; 2 School of Public Health, University of California–Berkeley, Berkeley, California, USA; 3 Northern California Cancer Center, Berkeley, California, USA; 4 Children’s Hospital of Central California, Madera, California, USA; 5 Battelle Memorial Institute, Columbus, Ohio, USA

**Keywords:** childhood cancer, dust, leukemia, organochlorine compounds, pesticides, polychlorinated biphenyls

## Abstract

**Background:**

Incidence of childhood leukemia in industrialized countries rose significantly during 1975–2004, and the reasons for the increase are not understood.

**Objectives:**

We used carpet dust as an exposure indicator to examine the risk of childhood leukemia in relation to residential exposure to persistent organochlorine chemicals: six polychlorinated biphenyl (PCB) congeners and the pesticides *α*- and *γ*-chlordane, *p*,*p*′-DDT (dichlorodiphenyltrichloroethane), *p*,*p*′-DDE (dichlorodiphenyldichloroethylene), methoxychlor, and pentachlorophenol.

**Methods:**

We conducted a population-based case–control study in 35 counties in northern and central California in 2001–2006. The study included 184 acute lymphocytic leukemia (ALL) cases 0–7 years of age and 212 birth certificate controls matched to cases by birth date, sex, race, and Hispanic ethnicity. We collected carpet dust samples from the room where the child spent the most time before diagnosis (similar date for controls) using a specialized vacuum.

**Results:**

Detection of any PCB congener in the dust conferred a 2-fold increased risk of ALL [odds ratio (OR) = 1.97; 95% confidence interval (CI), 1.22–3.17]. Compared with those in the lowest quartile of total PCBs, the highest quartile was associated with about a 3-fold risk (OR = 2.78; 95% CI, 1.41–5.48), and the positive trend was significant (*p* = 0.017). Significant positive trends in ALL risk were apparent with increasing concentrations of PCB congeners 118, 138, and 153. We observed no significant positive associations for chlordane, DDT, DDE, methoxychlor, or pentachlorophenol. The associations with PCBs were stronger among non-Hispanic whites than among Hispanics despite similar distributions of PCB levels among controls in each racial/ethnic group.

**Conclusions:**

Our findings suggest that PCBs, which are considered probable human carcinogens and cause perturbations of the immune system, may represent a previously unrecognized risk factor for childhood leukemia.

Childhood leukemia is the most common childhood cancer, and the etiology is poorly understood (Buffler et al. 2005; Ross and Spector 2006). Acute lymphocytic leukemia (ALL) accounts for about 80% of childhood leukemias in most Western countries (Dalmasso et al. 2005; Kroll et al. 2006; Ries et al. 2004); incidence peaks at 2–5 years of age, indicating that early-life exposures are important. Incidence of ALL is highest in industrialized countries (Ross and Spector 2006) and rose significantly over the period 1975–2004 in the United States, Europe, and Japan (Dalmasso et al. 2005; Kroll et al. 2006; Nishi et al. 1996; Ries et al. 2004), suggesting that environmental exposures or lifestyle changes may play an etiologic role.

Organochlorine insecticides [e.g., DDT (dichlorodiphenyltrichloroethane) and chlordane] and polychlorinated biphenyls (PCBs) became common environmental contaminants after World War II because of their widespread use, persistence in the environment, and bioaccumulation through the food chain. Because of concerns about detrimental effects on the environment and human health, uses of DDT, PCBs, and chlordane were banned in the United States in 1972, 1977, and 1988, respectively. However, these chemicals persist indoors in carpets, where they are protected from degradation by sunlight, moisture, and microorganisms. Ingestion of house dust is an important route of chemical exposure for young children, who spend most of their time indoors and frequently put their hands in their mouths (Bradman et al. 1997; Lanphear et al. 1996; Thornton et al. 1990; Wilson et al. 2001). Concentrations of organochlorines in serum, breast milk, and dietary sources have decreased substantially since the 1970s (Furst 2006; Schecter et al. 2005); as a result, indoor sources can be a major contributor to exposure for children living in older homes, where these chemicals are frequently detected.

Epidemiologic studies have implicated residential and parental exposure to pesticides as risk factors for childhood leukemia. However, specific pesticides were not identified in most studies, which relied primarily on self-reports about pesticide use. PCBs are considered probable human carcinogens and cause perturbations of the immune system (Hertz-Picciotto et al. 2008). PCB congeners commonly found in blood, adipose tissue, and house dust have been associated with increased risk of adult non-Hodgkin lymphoma (NHL) in cohort and case–control studies (Colt et al. 2005; De Roos et al. 2005; Engel et al. 2007). In this report, we evaluated the hypothesis that persistent organo-chlorine chemicals may increase the risk of childhood leukemia, and we used residential carpet dust as an indicator of exposure. No previously published population-based study has evaluated residential exposure to these chemicals and risk of childhood leukemia.

## Methods

### Study population

We conducted a population-based case–control study of childhood leukemia in northern and central California (Northern California Childhood Leukemia Study), which included 17 counties in the San Francisco Bay area and 18 counties in the Central Valley. As described previously (Chang et al. 2006; Ma et al. 2004, 2005), cases ≤ 14 years of age were rapidly ascertained from the nine major pediatric clinical centers in the study area, and controls, individually matched to cases on age, sex, race, Hispanic ethnicity, and maternal residence in the 35-county study area, were selected from California birth certificate files. A detailed in-home interview that included residential and parental occupational history (tier 1) was conducted with the child’s primary caretaker after consent was obtained. Beginning with cases diagnosed in December 1999 (and a similar reference date for controls), cases and controls ≤ 7 years of age who were living at the home they occupied at the time of diagnosis were eligible for a second interview (tier 2). During the eligibility period for tier 2 participation, the participation rate in the main study among families of cases < 8 years of age was 86%. Among households of potential controls, 9% could not be located and 21% refused before eligibility could be determined. Of the 606 controls determined to be eligible for the main study, 536 (88.5%) participated.

In the tier 2 interview, we obtained detailed information on home and garden pesticide use, inventoried pesticides in storage, and collected carpet dust samples. We limited eligibility to younger cases and controls so that the carpet dust sample would reflect exposures over a substantial portion of the child’s life. We also took a global positioning system measurement of the home location and determined whether the residence was located in an urban, suburban, or rural area based on the 2000 U.S. census block characteristics (U.S. Census Bureau 2002). We mapped crop fields within 1,500 m if the home was located in an agricultural area. Among 244 cases and 305 controls eligible for a tier 2 interview through March 2006, 225 cases (92%) and 244 controls (80%) participated. The primary reason for nonparticipation was refusal.

We collected dust samples using a specialized vacuum, the high-volume small-surface sampler (HVS3; Cascade Stack Sampling System, Venice, FL), from December 2001 through March 2006. Because of the longer time period involved in identifying, enrolling, and interviewing birth certificate controls in the main study, the time between the reference date and dust collection was less for cases {median [interquartile range (IQR)] years: cases, 0.96 (0.76–1.38); controls, 1.55 (1.24–1.97)}. As previously described (Colt et al. 2008), we asked parents to identify the room where the child had spent the most time, while awake, during the year before diagnosis or reference date. We took the HVS3 sample in that room if there was a carpet or area rug measuring at least 9 ft^2^ that was present before the reference date. Most samples were taken in the living room or family room. A total of 203 cases and 212 controls met the eligibility requirements for sampling and had adequate dust (~ 0.25 g) collected for analysis by at least one of the analytic methods (described below). A total of 22 cases and 32 controls either had no eligible carpet or the dust amount collected was too little for analysis. We present results for ALL (*n* = 184), which constituted 91% of the leukemia cases.

### Laboratory methods

Details of the carpet dust sample shipping, processing, and chemical analyses have been described previously (Colt et al. 2008). Briefly, we sieved dust samples and retained the fine fraction (< 150 μm). We used a hexane:acetone extraction method for *α*- and *γ*-chlordane (hereafter chlordane), *p*,*p*′-DDE (dichlorodiphenyldichloroethylene; hereafter DDE), *p*,*p*′-DDT (hereafter DDT), dieldrin, lindane, methoxychlor, and six PCB congeners (105, 118, 138, 153, 170, and 180). We used an acid herbicide extraction method to measure pentachlorophenol. Detection and quantification were by gas chromatography/mass spectrometry in the multiple ion detection mode. Quality control samples included duplicates, the same duplicate spiked with 250 ng of each analyte, and a solvent method blank. We spiked carbon-13–labeled surrogate recovery standards (SRS) representing all major classes of analytes into all samples before extraction to aid in identification and as a check on method performance. All sample batches contained 12 samples, including one duplicate and at least four case and four control samples; laboratory personnel were blind to case or control status.

Mean sample recoveries (without SRS correction) ranged from 85% for PCB-105 to 118% for methoxychlor. The relevant SRS recovery averages ranged from 82% to 111% in the quality control samples. Results were similar when we used SRS-corrected and -uncorrected concentrations; therefore, we report the uncorrected concentrations. Table 1 shows the method detection limits (DLs), detection frequencies, and distributions of organochlorine chemical concentrations among controls. Spearman rank correlations between concentrations of the PCB congeners were significant (*p* < 0.05) and ranged from 0.20 for PCB-105 and PCB-180 to 0.71 for PCB-153 and PCB-180. PCB congeners 118, 138, 153, and 180 were moderately correlated with chlordane, DDE, DDT, methoxychlor, and pentachlorophenol; correlations ranged from 0.18 for PCB-118 and methoxychlor to 0.40 for PCB-138 and methoxychlor.

### Statistical analysis

The organochlorine concentrations distributed log normally, and analyses were based on the natural log of the concentration, our primary exposure metric. We also calculated the chemical loading, an estimate of the amount of chemical per square meter of carpet, by multiplying the concentration of the chemical by the dust loading (amount of fine dust collected divided by the sampled area). The dust loadings were similar among cases and controls: cases, median, 0.8 g/m^2^ (IQR, 0.3–2.6 g/m^2^); controls, 1.1 g/m^2^ (IQR, 0.4–2.7 g/m^2^).

For chemicals that were detected in a minimum of 24% of samples, we used a single imputation method (Lubin et al. 2004) that selects a value from the modeled lognormal distribution to assign values for samples that were below the method DL. We used the LIFEREG procedure in SAS (version 9.1; SAS Institute Inc., Cary, NC) to derive chemical-specific models that included parameters significantly associated with the concentrations of the chemical in the house dust samples among controls; we used these predictive models to “fill in” imputed concentrations for measurements below the DL. Older homes had higher concentrations of all of the organochlorine chemicals; therefore, we included housing age in all imputation models. We included other factors significantly associated with concentrations of specific chemicals in the respective models: race/ethnicity for DDE (higher in Hispanic homes) and chlordane (lower in homes of non-Hispanic nonwhites); age for pentachlorophenol (lower in homes of children < 1 year of age compared with homes with children 5–7 years of age); year of the dust sampling (2001–2006) for DDE and chlordane (lower concentrations in later years); season of dust sampling for DDT and chlordane (lower concentrations in the summer and fall compared with winter); residence within 1,500 m of crops for DDE (higher compared with residences without crops within 1,500 m) and pentachlorophenol (lower compared with residences without crops within 1,500 m); maternal age for pentachlorophenol (higher in homes of mothers = 30 years of age); and whether the child was breast-fed > 6 months for the PCB congeners (higher compared with homes of children never breast-fed or breast-fed < 6 months).

For analyses of pentachlorophenol, chlordane, and total PCBs (sum of all congeners), we categorized the distribution into quartiles based on measured and imputed values among controls because few homes had no detections. For the other chemicals that were detected in > 20% of homes, the reference group was those with no detectable concentration, and we categorized detected concentrations by tertiles or the median of the detected concentration among controls. We calculated odds ratios (ORs) and 95% confidence intervals (CIs) using unconditional logistic regression. We conducted tests for trend by including the continuous variable (values below the DL were based on one imputation) in the regression models. We adjusted all analyses for age, sex, and race/ethnicity (non-Hispanic white, Hispanic, non-Hispanic other race), which were study matching factors. We also adjusted for confounding factors that resulted in changes to the ORs of ≥ 10%. For the PCBs these included the age of the home (built before 1980built before 1980 or later) and whether and for how long the mother breast-fed the child (never/< 6 months, ≥6 months). For the other organochlorines, we adjusted for income (< $30,000, $30,000–$59,000, ≥$60,000) and the year and season of the dust sampling. We evaluated potential effect modification by breast-feeding status and maternal age, because breast-feeding is a source of exposure to persistent organo-chlorine chemicals for infants, and older mothers are more likely to have higher serum concentrations.

## Results

Because of the study design, cases and controls who were eligible for the tier 2 interview had moved less frequently than all study participants < 8 years of age. This additional eligibility requirement and, to a lesser extent, refusals by some eligible participants resulted in some differences in sociodemographic factors between the tier 2 study population in these analyses and the main study participants < 8 years of age. Tier 2 participants included a greater percentage of children that were non-Hispanic whites (39% vs. 34% among cases; 48% vs. 41% among controls), a lower percentage of Hispanic children (39% vs. 46% among cases; 32% vs. 41% among controls), and a greater percentage of children from higher-income (≥ *$*60,000) households (41% vs. 35% among cases; 59% vs. 50% among controls). Tier 2 cases and controls did not differ significantly by sex, age, race/ethnicity, urbanicity of their residence, breast-feeding status and duration, or mother’s age at their birth (Table 2). Fewer controls than cases lived in households with incomes < $60,000, and more controls lived in single-family homes and were interviewed in the spring or fall. The duration of residence in the interview home before diagnosis/reference date was similar for cases and controls [median (IQR): cases, 2.5 years (1.5–3.8 years); controls, 2.4 years (1.6–4.1 years)] and represented a significant portion of the child’s lifetime for most cases and controls.

Detection of any of the measured PCB congeners in the dust conferred a 2-fold increased risk of ALL (OR = 1.97; 95% CI, 1.22–3.17). Compared with those in the lowest quartile of total PCBs, the highest quartile was associated with an almost 3-fold risk (OR = 2.78; 95% CI, 1.41–5.48), and the positive trend was significant (*p* = 0.017) (Table 3). Detections of four of the six measured PCB congeners (118, 138, 153, and 170) were each associated with significant or marginally significant elevated risk of ALL, with the highest risk observed for PCB-170 (OR = 2.05; 95% CI, 0.99–4.26). We found a significant positive trend in risk with concentrations of PCB-118 (*p* = 0.018), PCB-138 (*p* = 0.026), and PCB-153 (*p* = 0.017). When we measured exposure in terms of PCB loadings (nanograms per square meter), we observed no association with risk of ALL. ORs for some specific congeners were elevated but did not demonstrate significant positive trends with increasing loadings.

Increasing loadings of DDE and pentachlorophenol were associated with significant inverse trends in ALL risk. There were no other significant or noteworthy findings for the organochlorine pesticides. Risk estimates for the PCB congeners were not changed by adjustment for concentrations of the organo-chlorine pesticides. The moderate to high correlation between PCB congeners precluded mutual adjustment of the individual congeners for each other. When we excluded children 2–7 years of age who lived at their tier 2 home for < 1 year before the diagnosis or reference date, we obtained similar results for both the PCBs and organochlorine pesticides.

We observed consistently higher risks of ALL associated with detections of total PCBs and individual PCB congeners in carpet dust samples among non-Hispanic whites compared with Hispanics (Table 4). These differences were statistically significant for total PCBs and PCB-153 (*p*-value for interaction = 0.016 and 0.015, respectively). We observed similar results in analyses by concentration of total PCBs and individual congeners (data not shown). ORs for PCB loadings tended to be higher among non-Hispanic whites; however, as we observed for all racial/ethnic groups combined, we found no significant trends in either group (data not shown). We found no significant differences by ethnicity for concentrations or loadings of the organochlorine pesticides (data not shown).

**Figure 1:**
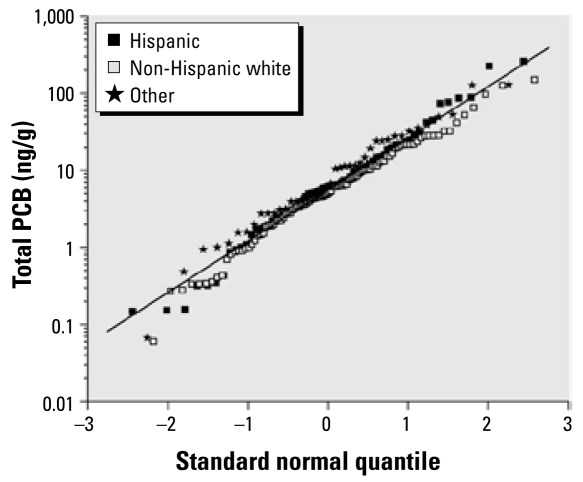
Quantile–quantile plot of summed PCB concentrations for Hispanic, non-Hispanic (NH) white, and other racial groups. The fitted line, which is based on all measurement data, represents log-normally distributed data with geometric mean of 5.63 ng/g, geometric SD of 4.65 ng/g, and arithmetic mean of 16.23 ng/g.

Among controls, the distribution of total PCBs was similar for Hispanics, non-Hispanic whites, and non-Hispanics of other races (Figure 1); thus, differences in household exposures are unlikely to explain the differences in observed risk. We evaluated potential confounding and effect modification by several factors that differed between Hispanics and non-Hispanic whites, the two largest ethnic groups. Hispanics reported fewer hours of child care attendance, shorter duration of breast-feeding, younger maternal age, lower income, and a greater number of children in the household compared with non-Hispanic whites. Adjustment for these factors in separate models by ethnicity did not substantially change the association with total PCBs in either group. We also we stratified our analyses by these factors. Among non-Hispanic white children, ORs increased with total PCB concentrations within all strata, whereas among Hispanics risk was elevated only among children of older mothers (> 30 years) and in households with only one or no other children. The ORs for individual PCB congeners showed a similar pattern (data not shown). Among all racial/ethnic groups combined, we found little evidence of effect modification by these factors (data not shown).

## Discussion

Our study is the first evaluation of the relationship between residential concentrations of PCBs and risk of ALL in children. We observed increased risk of ALL with increasing concentrations of total PCBs and with specific PCB congeners in dust samples taken from the room in which the child spent the most time. In contrast, dust levels of the persistent organochlorine pesticides DDT, DDE, chlordane, methoxychlor, and pentachlorophenol were not associated with increased risk. PCBs are probable human carcinogens and cause perturbations of the immune system, and concentrations in house dust and plasma have been associated with increased risk of adult NHL in epidemiologic studies (Colt et al. 2005; De Roos et al. 2005; Engel et al. 2007). Our findings suggest that residential exposure to PCBs may represent a previously unrecognized risk factor for the development of ALL in young children.

The associations we observed with specific PCB congeners were consistently stronger among non-Hispanic whites compared with Hispanics. We were unable to explain this effect modification, although unmeasured factors such as cultural or biological characteristics may have been responsible. It is also possible that residential dust samples were not an important source of exposure among Hispanic children in our study or that other unmeasured sources of exposure were more important, resulting in misclassification that would have obscured positive associations with risk. For example, Hispanic children may have spent more time away from their home in the care of relatives. We did not have information on the usual amount of time spent in the home or in the room where we conducted the dust sampling, which would have partly addressed this question.

Residential exposure to PCBs and childhood leukemia risk has not been evaluated previously in a population-based study. A small study comparing bone marrow concentrations of PCBs in 38 children with ALL and 15 healthy children selected from the same hospital found no significant differences in mean concentrations across the two sample pools (Scheele et al. 1992). That study was limited by a small size, measurement of PCBs in samples collected postdiagnosis, and use of a comparison group that may not have been representative of the general population. Case–control and cohort studies of adult NHL in the general population have shown consistent positive associations between PCB concentrations and risk (Engel et al. 2007). A population-based case–control study measuring PCBs in both house dust and plasma samples found increased NHL risk for both exposure assessment methods (Colt et al. 2005; De Roos et al. 2005), suggesting that house dust may be a good exposure indicator even in an older population.

We found no associations with childhood ALL for the organochlorine pesticides. Although self-reported parental occupational and residential exposures to pesticides have been associated with increased ALL risk in many studies (Buffler et al. 2005; Daniels et al. 1997; Infante-Rivard and Weichenthal 2007; Zahm and Ward 1998), nearly all of them lacked information on specific pesticide active ingredients. A study in Costa Rica (Monge et al. 2007) reported a nonsignificantly elevated risk of childhood leukemia associated with organochlorine pesticide use by the father during the pregnancy; however, results were based on small numbers, and ORs for specific pesticides were not presented. Among a cohort of agricultural pesticide applicators, paternal preconception use of aldrin was associated with an elevated risk of all childhood cancers; however, the numbers were too small to evaluate specific cancer types (Flower et al. 2004). Occupational exposure to chlordane has been linked to an elevated risk of adult leukemia in some studies (Brown et al. 1990; Purdue et al. 2007). In contrast to our findings, two case series reported blood dycrasias and leukemias after professional pest control treatments of residences with chlordane (Epstein and Ozonoff 1987; Infante et al. 1978), providing limited evidence that residential chlordane exposure may disrupt the immune system in humans.

Based on mechanistic studies, animal studies, and epidemiologic studies of cancer in adults, the International Agency for Research on Cancer and the U.S. Environmental Protection Agency consider PCBs to be probable human carcinogens (International Agency for Research on Cancer 1997; U.S. Environmental Protection Agency 2005). PCBs are thought to exert their carcinogenic effect through several possible mechanisms, depending on the specific congener and tumor site. These include binding to the aryl hydrocarbon receptor, promotion of cell proliferation or inhibition of apoptosis, and toxicity to immune cells (Levin et al. 2005; Safe 1993; Tryphonas 1994; Wolff et al. 1997). All of the PCB congeners that we measured have demonstrated immunotoxic effects (Harper et al. 1995; Wolff et al. 1997) and, except for PCB-105, were individually associated with elevated risk of ALL.

Children may be exposed to PCBs and persistent organochlorine pesticides *in utero*, through breast-feeding and other dietary sources, through inhalation, and through ingestion of house dust (Calabrese et al. 1989; Guvenius et al. 2003; Hooper et al. 2007; Wilson et al. 2001). Young children are estimated to ingest about 100 mg of dust per day (Davis et al. 1990). Levels of PCBs and other organochlorine chemicals in blood, breast milk, and dietary sources have decreased considerably since the early 1970s (Furst 2006; Laden et al. 1999; Schecter et al. 2005). Accordingly, exposure to environmental sources such as dust and soil via ingestion, inhalation, and dermal absorption might account for an increasingly large share of children’s exposure to these chemicals. A study of preschool children in the United States found that indoor air and dietary sources were responsible for most exposures to PCBs (Wilson et al. 2001). PCB concentrations in carpet dust may be a good predictor of indoor air concentrations because carpets act as a reservoir for continued volatilization, and chemicals absorbed to suspended particles can be inhaled (Butte and Heinzow 2002). Sources of PCBs in carpet dust, particularly in older homes, include paints, sealants, caulking, floor finishing products, and older light fixtures (Herrick et al. 2004, 2007; Rudel et al. 2008).

Although PCB concentrations in carpet dust were associated with increased leukemia risk, PCB loadings were not. The loading incorporates the concentration and amount of dust collected per area of carpet sampled (i.e., concentration multiplied by the total dust collected per square meter of carpet) and is postulated to be a more accurate indicator of exposure for small children (Bradman et al. 1997; Lanphear et al. 1996). However, the amount of dust collected is likely to reflect recent vacuuming practices, and a single measurement of dust loading would not be reliable if there is a large variation in dust loading within a house or over time. Thus, incorporating the dust loading into the exposure metric may have introduced random error and obscured concentration-based associations with risk.

Our exposure assessment method is a major strength of our study. We conducted environmental sampling to measure concentrations of persistent organochlorine chemicals in the room in which the child spent the most time. Although it is desirable to do so, prospective or pretreatment exposure measurements in blood samples are difficult to obtain because of the rarity of the disease and the very young age of most children. Residential carpet dust sampling provides an alternative exposure assessment method that allows identification of individual compounds and is not subject to recall bias. Organochlorine pesticides and PCBs are known to persist in carpets, where they are protected from degradation by sunlight, moisture, and microbes, and most homes had detectable levels of these chemicals in the dust samples. Other strengths of our study include the rapid case ascertainment, population-based selection of controls, and high participation rates for the tier 2 interview.

A limitation of our study was the moderate number of participants with HVS3 dust samples, particularly after stratifying by ethnicity, limiting our statistical power. Further, our study population was limited to those who had not moved since the diagnosis or reference date. If PCB and organochlorine concentrations were substantially different among those who were less residentially stable, our results may not be generalizable to the general population. Another limitation, common to many case–control studies, was the lower response rates among controls compared with cases. Our population-based controls were previously shown to be comparable with all eligible controls with respect to maternal history of fetal loss, birth weight, birth order, and time since last live birth (Ma et al. 2004). In the present analysis, cases and controls in all ethnic groups were significantly different with respect to household income. However, income was not associated with PCB concentrations and was not a confounder in our analyses. Moreover, analyses stratified by income showed similar associations between PCB concentrations and ALL risk, providing some assurance that our findings were not caused by selection bias. The fact that we did not observe associations for other persistent organochlorine chemicals provides additional evidence against selection bias.

The positive association observed for PCBs and ALL could have been attributed to other chemicals in the home that were highly correlated with PCBs. Although we were able to rule out confounding by the organochlorine pesticides measured in this study, we did not measure concentrations of polybrominated diphenyl ethers (PBDEs), which have become common environmental contaminants because of their increased use as fire retardants since the 1970s. Several studies (Bradman et al. 1997; Furst 2006; She et al. 2007), including a cohort study of Hispanic women with young children in Salinas County, California (Bradman 2007), found no correlation between PCB-153 and PBDE concentrations in maternal blood samples, suggesting different routes of exposure for these classes of chemicals. However, future analyses of residential concentrations of organochlorine chemicals, PBDEs, and other pesticides will be informative.

In summary, we observed an increasing risk of ALL associated with increasing residential concentrations of PCBs. PCBs are considered probable human carcinogens and cause perturbations of the immune system. We found no evidence of a relationship between ALL risk and exposure to DDT, DDE, chlordane, or pentachlorophenol. Additional studies are needed to further evaluate these highly suggestive findings.

## Figures and Tables

**Table 1:** Percent detections and concentrations (ng/g) of persistent organochlorine compounds measured in carpet dust, among controls (*n* = 212)^a^

Compound	Method DL (ng/g)	Percent > DL	Geometric mean (ng/g)^b^	Geometric SD	Arithmetic mean (ng/g)^b^	Arithmetic SD
Total PCBs	—	64.6	5.63	4.65	16.23	32.05
PCB-105	1	11.3	—	—	—	—
PCB-118	1	29.2	0.3	13.0	3.69	9.87
PCB-138	1	48.1	1.0	6.9	6.84	17.10
PCB-153	1	48.6	1.0	5.7	5.77	17.50
PCB-170	2	7.1	—	—	—	—
PCB-180	2	36.8	1.2	3.5	3.81	11.28
*α*-Chlordane	2	94.8	9.7	4.1	33.22	91.35
*γ*-Chlordane	2	95.3	11.3	3.9	35.94	90.72
DDE	2	81.6	9.4	4.3	23.82	42.93
DDT	10	56.6	16.0	7.9	78.82	169.49
Lindane	10	5.7	—	—	—	
Dieldrin	50	6.6	—	—	—	
Methoxychlor	10	23.6	36.4	4.2	31.34	183.88
Pentachlorophenol	5	93.9	77.0	3.2	199.27	707.87

a Dust samples taken with the HVS3 vacuum in the room where the child spent the most time.

b Calculated assuming a log normal distribution and using the LIFEREG procedure in SAS.

**Table 2:** Characteristics of ALL cases and controls with HVS3 dust samples collected in tier 2 of the Northern California Childhood Leukemia Study, 2001–2006 [no. (%)]

Characteristic	Cases (*n* = 184)	Controls (*n* = 212)	*p*-Value^a^
Sex			

Male	106 (57.6)	117 (55.2)	0.628
Female	78 (42.4)	95 (44.8)	

Age (years)			

0–1	4 (2.2)	9 (4.3)	0.338
> 1–2	23 (12.5)	27 (12.8)	
> 2–5	101 (54.9)	126 (59.7)	
> 5–7	56 (30.4)	50 (23.2)	
Age (mean ± SD)	4.0 ± 1.8	3.8 ± 1.8	

Race/ethnicity			

Hispanic	71 (38.6)	68 (32.1)	0.161
Non-Hispanic white	71 (38.6)	102 (48.1)	
Non-Hispanic other race	42 (22.8)	42 (19.8)	

Household income (US$)			

< 30,000	43 (23.4)	32 (15.1)	0.002
30,000–59,999	65 (35.3)	55 (25.9)	
≥ 60,000	76 (41.3)	125 (59.0)	

Age of the residence			

≥ 1980	93 (50.5)	116 (54.7)	0.449
1950–1979	38 (20.6)	44 (20.8)	
< 1950	25 (13.6)	31 (14.6)	
Unknown	28 (15.2)	21 (9.9)	

Residence type			

Single family	148 (80.4)	185 (87.3)	0.064
Townhouse/apartment/mobile home/other	36 (19.6)	27 (12.7)	

Residence location			

Urban	133 (72.3)	159 (75.0)	0.145
Suburban	27 (14.7)	19 (9.0)	
Rural	21 (11.4)	32 (15.1)	
Unknown	3 (2.0)	2 (1.0)	

Season of interview			

Winter	58 (31.5)	36 (17.0)	0.001
Spring	50 (27.2)	87 (41.0)	
Summer	47 (25.5)	43 (20.3)	
Fall	29 (15.8)	46 (21.7)	

Duration of breast-feeding			

Never breast-fed or < 6 months	87 (47.3)	93 (43.8)	0.095
≥ 6 months	97 (52.7)	117 (55.2)	
Unknown	0	2 (0.01)	

Maternal age at birth of child (years)			

< 30	85 (46.2)	106 (50.0)	0.450
≥ 30	99 (53.8)	106 (50.0)	

*p*-Value derived from chi-square tests for categorical variables; unknown values were excluded for testing case–control differences for residence location and breast-feeding duration.

**Table 3:** ORs (95% CIs) for ALL associated with concentrations and loadings of PCB congeners and organochlorine pesticides in carpet dust samples, 2001–2006^a^

Chemical concentration					Chemical loading				
Concentration (ng/g)	Cases	Controls	OR^b^ (95% CI)	*p*-Value for trend	Loading (ng/m^2^ )	Cases	Controls	OR^b^ (95% CI)	*p*-Value trend
Total PCBs^b^					Total PCBs^b^				

< 2.3	26	53	1.0		< 2.2	56	53	1.0	
2.3 to < 6.0	52	53	2.13 (1.14–3.97)		2.2 to < 7.1	38	53	0.73 (0.41–1.31)	
6.0 to < 15.5	50	53	2.17 (1.14–4.13)		7.1 to < 21.7	41	53	0.70 (0.39–1.25)	
15.5–434.1	56	53	2.78 (1.41–5.48)	0.017	≥ 21.7	47	53	0.82 (0.46–1.47)	0.923

PCB-105^b^					PCB-105^b^				

< DL	161	188	1.0		< DL	161	188	1.0	
> DL	23	24	1.10 (0.59–2.06)		> DL				
1 to < 9.9	18	12	1.69 (0.78–3.67)		< 7.6	11	12	0.99 (0.42–2.36)	
9.9–48.6	5	12	0.50 (0.17–1.48)	0.018	≥ 7.6	12	12	1.22 (0.52–2.86)	0.871

PCB-118^b^					PCB-118^b^				

< DL	118	150	1.0		< DL	118	150	1.0	
> DL	66	62	1.56 (0.98–2.48)		> DL				
1 to < 4.2	21	21	1.62 (0.81–3.24)		< 2.7	27	21	2.04 (1.06–3.92)	
4.2 to < 8.7	19	20	1.28 (0.63–2.60)		2.7 to < 9.8	19	20	1.26 (0.62–2.54)	
8.7–95.0	26	21	1.78 (0.91–3.47)	0.018	≥ 9.8	20	21	1.37 (0.68–2.79)	0.655

PCB-138^b^					PCB-138^b^				

< DL	77	109	1.0		< DL	77	109	1.0	
> DL	107	102	1.65 (1.07–2.53)		> DL				
1 to < 3.1	26	34	1.20 (0.66–2.21)		< 2.8	41	34	1.89 (1.07–3.32)	
3.1 to < 8.2	45	34	2.06 (1.18–3.59)		2.8 to < 11.8	36	34	1.63 (0.92–2.90)	
8.2–144.6	36	34	1.70 (0.93–3.10)	0.026	≥ 11.8	29	34	1.32 (0.71–2.44)	0.690

PCB-153^b^					PCB-153^b^				

< DL	81	109	1.0		< DL	81	109	1.0	
> DL	103	103	1.67 (1.06–2.63)		> DL				
1.1 to < 2.8	32	34	1.41 (0.77–2.60)		1 to < 2.6	45	34	2.46 (1.36–4.42)	
2.8 to < 6.2	35	34	1.45 (0.80–2.64)		2.6 to < 9.0	26	34	1.15 (0.62–2.16)	
6.2–176.4	36	35	1.60 (0.88–2.89)	0.018	≥ 9.0	31	35	1.42 (0.76–2.65)	0.656

PCB-170^b^									

< DL (< 2)	162	197	1.0						
> DL (2–67.9)	22	15	2.05 (0.99–4.26)	—					

PCB-180^b^					PCB-180^b^				

< DL	109	134	1.0		< DL	109	134	1.0	
> DL	75	78	1.30 (0.82–2.05)		> DL				
2 to < 3.0	28	26	1.36 (0.73–2.53)		< 2.7	30	26	1.73 (0.92–3.26)	
3.0 to < 6.1	17	26	0.92 (0.45–1.87)		2.7 to < 9.5	17	26	0.80 (0.40–1.61)	
6.1–107.8	30	26	1.59 (0.84–3.01)	0.086	≥ 9.5	28	26	1.44 (0.77–2.72)	0.191

α-Chlordane^c^					α-Chlordane^c^				

< 3.5	40	53	1.0		< 3.3	59	53	1.0	
3.5 to < 8.3	44	53	1.18 (0.60–2.09)		3.3 to < 10.8	43	53	0.67 (0.37–1.21)	
8.3 to < 22.9	49	53	1.32 (0.71–2.43)		10.8 to < 30.4	24	53	0.34 (0.18–0.66)	
22.9–1,916	51	53	1.27 (0.69–2.35)	0.098	≥ 30.4	56	53	0.78 (0.42–1.42)	0.286

DDE^c^					DDE^c^				

< DL	39	39	1.0		< DL	39	39	1.0	
> DL	145	173	0.87 (0.51–1.50)		> DL				
2 to < 9.4	38	58	0.74 (0.39–1.41)		2 to < 8.0	53	58	1.10 (0.59–2.07)	
9.4 to < 21.7	59	57	1.08 (0.58–2.02)		8.0 to < 30.7	43	57	0.78 (0.41–1.48)	
21.7–850.4	48	58	0.83 (0.43–1.59)	0.794	≥ 30.7	47	58	0.69 (0.36–1.34)	0.021

DDT^c^					DDT^c^				

< DL	86	92	1.0		< DL	86	92	1.0	
> DL	98	120	0.86 (0.56–1.32)		> DL				
10 to < 38.2	34	40	0.85 (0.47–1.52)		< 34.6	41	40	1.12 (0.64–1.97)	
38.2 to < 117.8	29	40	0.77 (0.42–1.41)		34.6 to < 164.2	25	40	0.72 (0.38–1.36)	
117.8–17,310.	35	40	0.95 (0.53–1.69)	0.709	≥ 164.2	32	40	0.73 (0.40–1.32)	0.165

Methoxychlor^c^					Methoxychlor^c^				

< DL	150	162	1.0		< DL	150	162	1.0	
> DL	34	50	0.79 (0.47–1.33)		> DL				
10 to < 18.5	5	17	0.40 (0.14–1.20)		10 to < 23.1	8	17	0.67 (0.26–1.74)	
18.5 to < 61.6	18	16	1.22 (0.58–2.55)		23.1 to < 72.8	10	16	0.77 (0.32–1.81)	
61.6–2360	11	17	0.69 (0.29–1.62)	0.549	≥ 72.8	16	17	0.90 (0.42–1.94)	0.325

Pentachlorophenol^c^					Pentachlorophenol^c^				

< 32.2	38	49	1.0		< 32.7	51	49	1.0	
32.2 to < 75.8	46	50	1.28 (0.68–2.40)		32.7 to < 82.2	34	50	0.56 (0.29–1.08)	
75.8 to < 164.7	47	50	1.46 (0.78–2.74)		82.2 to < 272.5	43	50	0.78 (0.42–1.47)	
164.7–22,676	31	50	0.84 (0.43–1.65)	0.476	≥ 272.5	32	50	0.47 (0.24–0.92)	0.045

a Carpet dust samples taken from the room where the child spent the most time while awake.

b ORs adjusted for age, sex, race/ethnicity, age of home, and breast-feeding duration.

c ORs adjusted for age, sex, race/ethnicity, income, year, and season of the interview/dust collection.

**Table 4:** ORs (95% CIs) for childhood leukemia associated with detections of total PCBs and individual PCB congeners in the home, for non-Hispanic whites and Hispanics^a^

	Non-Hispanic white			Hispanic		
Compound	Cases (*n* )	Controls (*n* )	OR^b^ (95% CI)	Cases (*n* )	Controls (*n*)	OR^b^ (95% CI)
Any PCB						

All < DL	7	40	1.0	20	21	1.0
Any > DL	64	62	6.3 (2.5–16.0)	51	47	1.15 (0.54–2.44)

PCB-105						

< DL	60	93	1.0	62	56	1.0
> DL	11	9	2.01 (0.78–5.21)	9	12	0.71 (0.27–1.89)

PCB-118						

< DL	38	71	1.0	51	50	1.0
> DL	33	31	1.97 (1.02–3.77)	20	18	0.98 (0.44–2.19)

PCB-138						

< DL	24	52	1.0	32	36	1.0
> DL	47	49	2.03 (1.07–3.87)	39	32	1.39 (0.68–2.80)

PCB-153						

< DL	17	54	1.0	40	36	1.0
> DL	53	49	3.66 (1.80–7.44)	31	32	0.84 (0.41–1.73)

PCB-170						

< DL	60	95	1.0	63	64	1.0
> DL	11	7	2.76 (0.97–7.80)	8	4	1.91 (0.51–7.10)

PCB-180						

< DL	32	39	1.0	44	48	1.0
> DL	39	40	1.84 (0.98–3.46)	27	20	1.52 (0.73–3.14)

a Carpet dust samples taken from the room where the child spent the most time while awake.

b ORs adjusted for age, sex, race/ethnicity, age of home, and breast-feeding duration.
